# In-school adolescents’ loneliness, social support, and suicidal ideation in sub-Saharan Africa: Leveraging Global School Health data to advance mental health focus in the region

**DOI:** 10.1371/journal.pone.0275660

**Published:** 2022-11-09

**Authors:** Richard Gyan Aboagye, Bright Opoku Ahinkorah, Abdul-Aziz Seidu, Joshua Okyere, James Boadu Frimpong, Manasi Kumar

**Affiliations:** 1 Department of Family and Community Health, Fred N. Binka School of Public Health, University of Health and Allied Sciences, Hohoe, Ghana; 2 School of Public Health, Faculty of Health, University of Technology Sydney, Sydney, Australia; 3 Department of Population and Health, University of Cape Coast, Cape Coast, Ghana; 4 Department of Estate Management, Takoradi Technical University, Takoradi, Ghana; 5 College of Public Health, Medical and Veterinary Sciences, James Cook University, Douglas, Australia; 6 Department of Health, Physical Education, and Recreation, University of Cape Coast, Cape Coast, Ghana; 7 Department of Kinesiology, New Mexico State University, Las Cruces, NM, United States of America; 8 Brain and Mind Institute, Aga Khan University, Nairobi, Kenya; Universidade Federal do Rio Grande do Sul, BRAZIL

## Abstract

**Introduction:**

Adolescent and youth mental health problems are increasingly becoming an area of concern in global health. Young people in sub-Saharan Africa experience significant adversities and systemic challenges despite technological advancements and demographic transition that the region is experiencing. We examined the nexus between experiences of loneliness, low social support, and presence of suicidal ideation among in-school adolescents in sub-Saharan Africa.

**Method:**

A total of 19,119 in-school adolescents from eight countries in sub-Saharan Africa were included in this study. Suicidal ideation was the main outcome variable and loneliness, and social support were the explanatory variables. Percentages were used to summarise the prevalence of suicidal ideation, loneliness, and social support among the in-school adolescents. A multivariable binary logistic regression analysis was later used to determine the association between suicidal ideation and the explanatory variables and covariates using Stata v16. Four models were tested using the regression analysis. We presented the regression results using adjusted odds ratios (aOR), with their respective 95% confidence intervals (CIs).

**Results:**

Overall, the past year prevalence of loneliness, peer support, one or more close friends, and suicidal ideation were 10%, 33.4%, 90.1%, and 14.5%, respectively. In-school adolescents who felt lonely (aOR = 1.88, 95% CI = 1.69, 2.09) were more likely to experience suicidal ideation. However, those who received peer support (aOR = 0.89, 95% CI = 0.82, 0.97) and had one or more close friends (aOR = 0.77, 95% CI = 0.68, 0.86) were less likely to experience suicidal ideation.

**Conclusion:**

These results point to the significant roles of loneliness, and lack of social support, in understanding suicidal ideations. Countries in sub-Saharan Africa need to improve child and adolescent mental health policies and programmes to respond to these risk factors and mental health challenges. Programmes with a differential focus on the needs of males and females, younger and older adolescents will be important in the future.

## Introduction

Rapid changes in society, family structure, and socioeconomic factors are contributing to severe adversities and stress in young people [1]. Self-harm and suicide rates have been going up in young populations prompting an examination of factors that trigger this behaviour [2]. Globally, it is reported that each year, more than 700,000 people die due to suicide and suicide is the fourth leading cause of death in 15–19 year old [3]. Additionally, suicide contributes to 1.4 percent of the global burden of disease and also emerged as the second cause of death among young people aged 15–29 years [4]. Hence, making suicide and concomitant issues of loneliness, isolation and poor support important public health concerns [5]. The African annual prevalence of suicide was found to be 34,000 with incidence of 3.2 per 100,000 [6]. A recent systematic review found that the median point prevalence of suicidal ideation in adolescents aged 10–19 from several countries in sub-Saharan Africa (SSA) was 11.6% [7]. It is noteworthy suicide does not occur sporadically; rather, it operates through a process which usually constitutes suicidal ideation and suicidal attempt [8]. Prior exposure to suicide and prior exposure to suicide attempts in the general population are associated with increased odds of subsequent suicidal behaviour [9]. Therefore, understanding suicidal ideation provides an enabling framework to tackle suicides in the general population.

In its generic form, suicidal ideation simply refers to when an individual thinks, conceives, and conceptualizes the processes to kill themselves for reasons known to them. Suicidal ideation can also be defined as “any self-reported passive thought about wanting to be dead or active thoughts about killing oneself not accompanied by preparatory behaviour” [10, p. 1]. It is estimated that the global lifetime prevalence of suicidal ideation ranges between 3.1 and 56 percent [11, 12]. Nonetheless, this prevalence might be understated as suicide is a sensitive topic, and is therefore rarely discussed openly in the society [13]. Among European countries, the lifetime prevalence of suicidal ideation among adolescents aged 15–16 years was 31.5 percent in Hungary and 15 percent in Armenia [14]. In a study conducted in Tanzania, Dunlavy et al. [15] found that among adolescents aged 11–16 years, seven percent of them reported suicidal ideation. However, in Ethiopia, a reported 23.7 percent of in-school adolescents reported suicidal ideation [16].

Articulating the pathway through which suicidal ideation operates is a challenging endeavor given the fact that it is affected by a multiplicity of factors which can broadly be categorized in biological (sex and age), psychological (affective disorder, major depression, and anxiety disorders) and environmental factors [17, 18]. Furthermore, literature shows that psychosocial triggers are one of the strongest predictors of suicidal ideation [11]. Among these psychosocial triggers are the experiences of loneliness and poor social support.

Loneliness is defined as the “cognitive discrepancy between the social relations an individual wishes to have and those one perceives to have and the affective reactions of sadness and emptiness resulting from this discrepancy” [19, p.133]. Evidence available shows that loneliness has adverse effects on the health of adolescents [20, 21]. Protracted or intense loneliness can easily become a trigger for suicidal ideation and other self-harming behaviours. For example, in a study among HIV/AIDS patients in Africa, loneliness and social support were significant predictors of suicidal ideation and attempt [22, 23]. Although Yadegarfard et al.’s [14] study was conducted among HIV/AIDS patients, which included adolescents, their findings suggest the potential association between loneliness, social support, and suicidal ideation among adolescents in the general population.

There is an urgent need to study mental health outcomes in low-resource settings like SSA because of the high unmet mental health needs due to unavailable human and financial resources [24]. Notwithstanding, there is a paucity of empirical evidence in SSA to substantiate the hypothesis that loneliness, and low social support increase the risk of suicidal ideation in adolescents. This presents a significant gap in the existing scientific discourse and evidence on correlates of adolescent suicidal ideation. The present study, therefore, seeks to test this hypothesis by investigating the association between loneliness, social support, and suicidal ideation among adolescents in SSA. Our findings provide important evidence which can facilitate targeted support and services to the most vulnerable sub-populations, as well as engineer countries in SSA towards the attainment of the Sustainable Development Goal (SDGs) 3, target 3.4 that points to self-harm and suicide prevention as key adolescent health strategies to be scaled at the population level.

We premise our study on the interpersonal-psychological theory of suicide (IPTS). This theory was developed by Joiner with the goal of providing some theoretical model of suicide behaviour [25]. According to the IPTS, suicidal desires and intentions stem from two main factors: perceived burdensomeness and a sense of low belongingness [26]. Joiner [27] describes the perceived burdensomeness as a situation whereby an individual feels or thinks that their existence is a burden for their friends and family as well as the larger society. Such sense of burdensomeness leads to a situation of self-hate and feelings of being a liability rather than an asset to everyone [25]. The second tenet of the IPTS is the low sense of belongingness [27]. This is the crust of our study. Low sense of belongingness denotes the feelings of being alienated from one’s family or social group, and thus, comprises loneliness and absence of reciprocal care [25, 27]. Our study perfectly fits into this IPTS as it shows how loneliness, and the absence of reciprocal care (i.e., social support) can lead to low sense of belongingness and consequently increase the risk of suicidal desires and ideation among adolescents. This relationship has been tested in previous studies that have found a statistically significant association between perceived burdensomeness and suicidal ideation, as well as between low sense of belongingness and suicidal ideation [28, 29].

## Methods and methods

### Data source and study design

We analyzed data from the Global School-based Student Health Survey (GSHS) of eight countries in SSA. Only the most recent surveys in the countries with dataset on the variables of interest were included in the study. The study countries and their survey years include Liberia (2017), Mauritius (2017), Benin (2016), Mozambique (2015), Seychelles (2015), Tanzania (2014), Namibia (2013), and Ghana (2012). The GSHS is a representative survey conducted among adolescents in World Health Organization (WHO) countries in partnership with the WHO, Center for Disease Control and Prevention (CDC), and Middle Tennessee State University (MTSU). In the respective study countries, the survey was conducted with support from the Ministries or Agencies in charge of Health and Education. The survey adopted a descriptive cross-sectional design. Structured self-administered questionnaires were used to collect data from the respondents. The dataset is freely available at https://extranet.who.int/ncdsmicrodata/index.php/catalog/GSHS.

### Sampling method and sample size

The respondents were selected using a two-stage cluster sampling technique. The first stage involved the selection of study schools with probability proportional to the school’s enrolment size. Later, classes with the study schools were randomly selected and within those schools, all the eligible students aged 10–19 years were included in the study. Among the selected students, only those who assented and their parents consented were given questionnaires to complete. In the GSHS, numerical weights were applied to each respondent to enable the generalization of the findings. A total of 19,119 adolescents from the eight countries in SSA were included in the study (Table 1).

**Table 1:** Distribution of the study sample.

Country	Year of survey	Sample at design	Weighted sample included in the study	Weighted percentage of sample included in the study
Benin	2016	2,536	2,127	11.1
Ghana	2012	3,632	2,889	15.1
Liberia	2017	2,744	1,594	8.3
Mauritius	2017	3,012	2,493	13.0
Mozambique	2015	1,918	1,339	7.0
Namibia	2013	4,531	3,603	18.9
Seychelles	2015	2,540	1,960	10.3
Tanzania	2014	3,793	3,114	16.3
All countries	2012–2017	24,706	19,119	100.0

### Variables

#### Outcome variable

Suicidal ideation was the outcome variable in this study. With this variable, the respondents were asked the question *“During the past 12 months*, *did you ever seriously consider attempting suicide*?*”*. The response categories were 1 = yes and 2 = no. This was further recoded into 0 = no and 1 = yes for this study’s purpose. The recoding employed in this study was informed by literature that used the GSHS dataset [30–32].

#### Key explanatory variables

We considered loneliness and social support as the key explanatory variables in this study. In assessing loneliness, the respondents were asked *“During the past 12 months*, *how often have you felt lonely*?*”*. The response options *were 1 = never*, *2 = rarely*, *3 = sometimes*, *4 = most of the time to 5 = always*. We dichotomised the responses into “no (those that responded never, rarely, and sometimes)” and “yes (for those that responded most of the time and always)”. Two variables, namely; peer support and having close friends were used to measure social support. Peer support was assessed using the question *“During the past 30 days*, *how often were most of the students in your school kind and helpful*?*”*. The response options were *1 = never*, *2 = rarely*, *3 = sometimes*, *4 = most of the time to 5 = always*. Those who responded never, rarely, and sometimes were recoded as “no (did not have peer support)” whilst those who responded most of the time and always were coded as “yes (had peer support)”. For close friends, the respondents were asked *“How many close friends do you have*?*”*. The responses were *1 = 0 friend; 2 = 1 friend; 3 = 2 friends; and 4 = 3 or more friends*. We recoded these response options into “1 = 0 friends” and “2 = 1 or more friends”.

#### Covariates

We included twelve variables as covariates in this study. These variables were selected based on their significant associations with suicide ideation from literature [30–34] and their availability in the GSHS dataset. The variables include the age of the respondent, sex, felt hungry, anxiety, current alcohol use, smoking cigarettes, tobacco use, marijuana use, parental or guardian supervision, parental or guardian bonding, parental or guardian connectedness, and parental or guardian respect for privacy. The detailed question, response options, and coding of these variables can be found in the supplementary file attached (S1 Table).

### Statistical analyses

**Fig 1:**
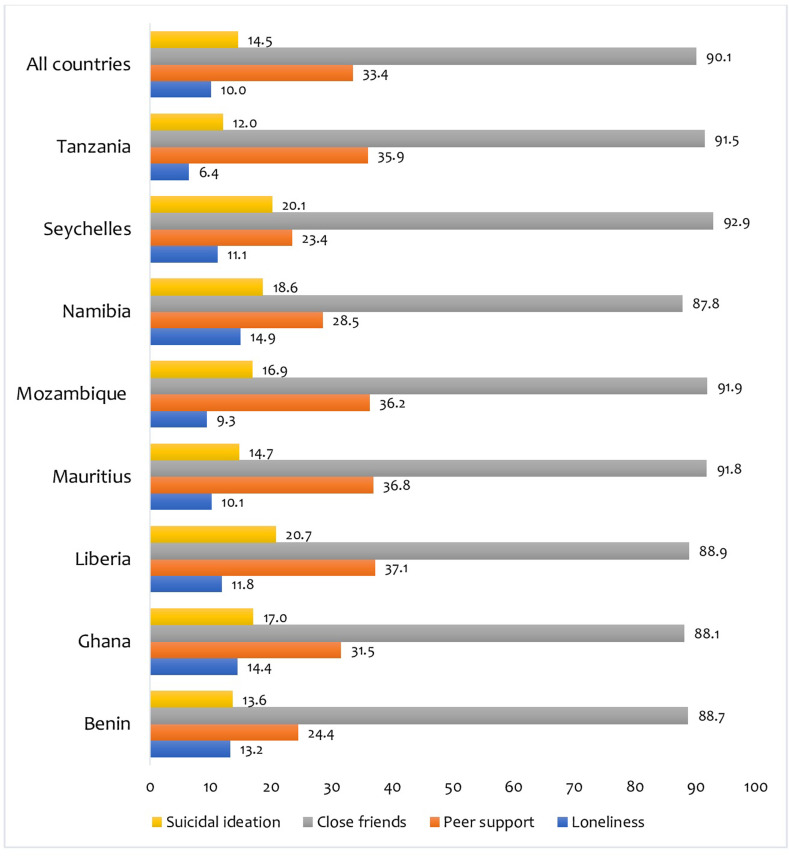
Prevalence of loneliness, peer support, close friends, and suicidal ideation among in-school adolescents.

We analysed the data using Stata software version 16.0 (Stata Corporation, College Station, TX, USA). Percentages were used to present the results of the prevalence of suicidal ideation, loneliness, and social support (Fig 1). We computed the distribution of the study sample per country across the variables used. Next, we examined the association between loneliness and social support and suicidal ideation using a mixed-effect multivariable binary logistic regression. Three models (Model O—Model II) were built to examine this association. Model O was fitted to contain only the outcome variable with no explanatory variables or covariates. Model I had only the key explanatory variables (loneliness and social support). Model II on the other hand contained the key explanatory variables and the covariates. The results were presented using adjusted odds ratios (aORs) with their respective 95% confidence interval (CIs). Statistical significance was set at p<0.05. We used the Stata command “melogit” in fitting the three models. We used the Akaike’s Information Criterion (AIC) tests to compare the fitness of the model with the last model being the best fitted model because of the least value of the AIC. Later, we examined the country-level association between the key explanatory variables and suicidal ideation using the Stata command “bysort” (Table 4). All the analyses were weighted. We applied a complex sample analysis (svy) and the inherent sample weight in all analyses we performed to reduce bias from non-response and improve generalisability to all in-school adolescents in sub-Saharan Africa. This manuscript was drafted by relying on the “Strengthening the Reporting of Observational Studies in Epidemiology” (STROBE) statement writing the manuscript [35].

### Ethical consideration

We did not seek ethical approval for the conduct of this study because the dataset was freely available in the public domain. However, prior to the start of the survey, institutional review approval was sought from the Institutional Review Board at MTSU. Also, permissions were sought from the Ministries of Education and Health in study countries via CDC office. All ethical guidelines concerning the use of human subjects especially minors were strictly adhered to. For the respondents aged below 18 years, written parental or guardian consent and child assent were sought from them before included in the study whilst only written consent were sought from those aged 18 years and above.

## Results

### Prevalence of loneliness, social support, and suicidal ideation among in-school adolescents in sub-Saharan Africa

Fig 1 shows the prevalence of loneliness, peer support, number of close friends, and suicidal ideation among in-school adolescents in the selected countries in SSA. The study found that the prevalence of loneliness, peer support, one or more close friends, and suicidal ideation were 10%, 33.4%, 90.1%, and 14.5%, respectively. Namibia recorded the highest (14.9%) prevalence of loneliness while Tanzania had the least (6.4%). Peer support and close friends were highest among adolescents from Liberia (37.1%) and Seychelles (92.9%), respectively, whereas the lowest were reported in Seychelles (23.4%) and Namibia (87.8%) accordingly. While Liberia reported the highest (20.7%) prevalence of suicidal ideation among in-school adolescents, Tanzania recorded the least (12.0%).

### Distribution of the covariates across the individual countries in sub-Saharan Africa

Table 2 shows the covariates across the individual countries in sub-Saharan Africa.

**Table 2:** Distribution of the covariates in each individual country.

Variable	Benin	Ghana	Liberia	Mauritius	Mozambique	Namibia	Seychelles	Tanzania
**Age group (years)**								
14 years or younger	269 (12.7)	854 (29.6)	261 (16.4)	1055 (42.3)	384 (28.7)	890 (24.7)	1191 (60.8)	1863 (59.8)
15 years or older	1858 (87.3)	2035 (70.4)	1333 (83.6)	1438 (57.7)	955 (71.3)	2713 (75.3)	769 (39.2)	1251 (40.2)
**Sex**								
Female	559 (26.3)	1403 (48.6)	747 (46.9)	1385 (55.6)	623 (46.5)	1958 (54.3)	1061 (54.1)	1607 (51.6)
Male	1568 (73.7)	1486 (51.4)	847 (53.1)	1108 (45.4)	716 (53.5)	1645 (45.7)	899 (45.9)	1506 (48.4)
**Felt hungry**								
No	1730 (81.3)	2488 (86.1)	1369 (85.9)	2327 (93.4)	1215 (90.8)	3290 (91.3)	1748 (89.2)	2939 (94.4)
Yes	397 (18.7)	401 (13.9)	225 (14.1)	166 (6.6)	124 (9.2)	313 (8.7)	212 (10.8)	175 (5.6)
**Anxiety**								
No	1692 (79.6)	2520 (87.2)	1299 (81.5)	2289 (91.8)	1222 (91.3)	3080 (85.5)	1752 (89.4)	2951 (94.8)
Yes	435 (20.4)	369 (12.8)	295 (18.5)	204 (8.2)	117 (8.7)	523 (14.5)	208 (10.6)	163 (5.2)
**Current alcohol use**								
No	1205 (56.7)	2513 (87.0)	1273 (79.9)	1878 (75.3)	1189 (88.8)	2391 (66.4)	1046 (53.4)	3019 (96.9)
Yes	922 (43.3)	376 (13.0)	321 (20.1)	615 (24.7)	150 (11.2)	1212 (33.6)	914 (46.6)	95 (3.1)
**Current cigarette smoking**								
No	2012 (94.6)	2740 (94.8)	1503 (94.3)	2110 (84.6)	1317 (98.3)	3292 (91.4)	1633 (83.3)	3032 (97.4)
Yes	115 (5.4)	149 (5.2)	91 (5.7)	383 (15.4)	22 (1.7)	311 (8.6)	327 (16.7)	82 (2.6)
**Current marijuana use**								
No	2104 (98.9)	2763 (95.6)	1509 (94.7)	2359 (94.6)	1327 (99.1)	3451 (95.8)	1827 (93.2)	3078 (98.8)
Yes	23 (1.1)	126 (4.4)	85 (5.3)	134 (5.4)	12 (0.9)	152 (4.2)	133 (6.8)	36 (1.2)
**Current tobacco use**								
No	2032 (95.5)	2641 (91.4)	1490 (93.5)	2271 (91.1)	1311 (97.9)	3398 (94.3)	1854 (94.6)	3040 (97.6)
Yes	95 (4.5)	248 (8.6)	104 (6.5)	222 (8.9)	28 (2.1)	205 (5.7)	106 (5.4)	74 (2.4)
**Parent or guardian supervision**								
No	1371 (64.4)	1676 (58.0)	827 (51.9)	1704 (68.4)	680 (50.8)	2183 (60.6)	1139 (58.1)	1290 (41.4)
Yes	756 (35.6)	1213 (42.0)	767 (48.1)	789 (31.6)	659 (49.2)	1420 (39.4)	821 (41.9)	1824 (58.6)
**Parent or guardian connectedness**								
No	1446 (68.0)	1821 (63.0)	899 (56.4)	1435 (57.6)	670 (50.0)	2139 (59.4)	1327 (67.7)	1886 (60.6)
Yes	681 (32.0)	1068 (37.0)	695 (43.6)	1058 (42.4)	669 (50.0)	1464 (40.6)	633 (32.3)	1228 (39.4)
**Parent or guardian bonding**								
No	1455 (68.4)	1807 (62.5)	963 (60.4)	1348 (54.1)	803 (60.0)	2434 (67.6)	1152 (58.8)	1886 (60.6)
Yes	672 (31.6)	1082 (37.5)	631 (39.6)	1145 (45.9)	536 (40.0)	1169 (32.4)	808 (41.2)	1228 (39.4)
**Parent or guardian respect for privacy**								
No	331 (15.5)	1214 (42.0)	826 (51.8)	792 (31.8)	251 (18.7)	1524 (42.3)	605 (30.9)	702 (22.5)
Yes	1796 (84.5)	1675 (58.0)	768 (48.2)	1701 (68.2)	1088 (81.3)	2079 (57.7)	1355 (69.1)	2412 (77.5)

Overall, there were many older in-school adolescents in the countries considered in this study. The distribution of sex varied from one country to the other. Most of the in-school adolescents in the studied countries did not feel hungry, were not anxious, did not use alcohol, cigarette, marijuana, and tobacco. Apart from in-school adolescents in Tanzania, the rest of the in-school adolescents did not receive parent or guardian supervision. In general, there was no parental or guardian connectedness and bonding. However, most of the in-school adolescents had respect for privacy from their parents or guardians.

### Association between loneliness, peer support, close friends, and suicidal ideation

Table 3 shows the results of the association between loneliness, peer support, close friends, and suicidal ideation among in-school adolescents. The study found that in-school adolescents who felt lonely (aOR = 1.88, 95% CI = 1.69, 2.09) were more likely to experience suicidal ideation. However, in-school adolescents who had peer support (aOR = 0.89, 95% CI = 0.82, 0.97) and those who had one or more close friends (aOR = 0.77, 95% CI = 0.68, 0.86) had lower odds of having suicidal ideation.

**Table 3:** Mixed effect analysis of the association between loneliness, peer support, close friends and suicidal ideation among the in-school adolescents.

Variables	Model O	Model I	Model II
		aOR [95% CI]	aOR [95% CI]
**Fixed effect results**			
**Felt lonely**			
No		1.00	1.00
Yes		2.49*** [2.25, 2.75]	1.88*** [1.69, 2.09]
**Peer support**			
No		1.00	1.00
Yes		0.84*** [0.78, 0.92]	0.89* [0.82, 0.97]
**Close friends**			
No		1.00	1.00
Yes		0.76*** [0.68, 0.85]	0.77*** [0.68, 0.86]
**Age group (years)**			
11–14			1.00
15–19			1.08 [0.99, 1.18]
**Sex**			
Female			1.00
Male			0.77*** [0.71, 0.83]
**Felt hungry**			
No			1.00
Yes			1.33*** [1.18, 1.49]
**Anxiety**			
No			1.00
Yes			1.95*** [1.76, 2.17]
**Current cigarette smoking**			
No			1.00
Yes			1.62*** [1.39, 1.89]
**Current tobacco use**			
No			1.00
Yes			1.33** [1.12, 1.58]
**Current alcohol use**			
No			1.00
Yes			1.30*** [1.18, 1.42]
**Current marijuana use**			
No			1.00
Yes			1.24* [1.01, 1.52]
**Parent or guardian supervision**			
No			1.00
Yes			1.04 [0.96, 1.14]
**Parent or guardian connectedness**			
No			1.00
Yes			0.82*** [0.75, 0.90]
**Parent or guardian bonding**			
No			1.00
Yes			0.92 [0.84, 1.01]
**Parent or guardian respect for privacy**			
No			1.00
Yes			0.79*** [0.73, 0.86]
**Random effect results**			
Primary sampling unit variance (95% CI)	0.020 [0.008, 0.046]	0.017 [0.007, 0.044]	0.014 [0.005, 0.040]
Intra-Class Correlation	0.006	0.005	0.004
Likelihood ratio test	13.51 (<0.001)	10.75 (<0.001)	7.77 (0.003)
Wald chi-square	Reference	374.60 (<0.001)	877.56 (<0.001)
**Model fitness**			
Log-likelihood	-8614.7636	-8441.1852	-8177.9355
Akaike Information Criterion	17233.53	16892.37	16389.87
Total sample	19,119	19,119	19,119
Number of clusters	122	122	122

* *p* < 0.05,

** *p* < 0.01,

*** *p* < 0.001

*Model I consisted of only the key explanatory variables

*Model II was adjusted for the covariates

### Association between loneliness, peer support, close friends, and suicidal ideation by country

Table 4 shows the results of the association between loneliness, social support, close friends and suicidal ideation segregated by country. The study found that in-school adolescents who felt lonely and lived in Benin (aOR = 1.97, 95% CI = 1.42, 2.74); Ghana (aOR = 1.53, 95% CI = 1.19, 1.98); Mauritius (aOR = 2.68, 95% CI = 1.91, 3.75); Namibia (aOR = 1.57, 95% CI = 1.25, 1.96); Seychelles (aOR = 4.09, 95% CI = 2.97, 5.64); and Tanzania (aOR = 1.94, 95% CI = 1.32, 2.85) were more likely to experience suicidal ideation. Conversely, in-school adolescents who had close friends and were from Mozambique (aOR = 0.50, 95% CI = 0.29, 0.85) and Tanzania (aOR = 0.42, 95% CI = 0.30, 0.58) were less likely to experience suicidal ideation.

**Table 4:** Association between loneliness, social support and suicidal ideation segregated by country.

Variable	Model I	Model II
	aOR [95% CI]	aOR [95% CI]
**Benin**		
**Felt lonely**		
No	1.00	1.00
Yes	2.50*** [1.83, 3.41]	1.97*** [1.42, 2.74]
**Peer support**		
No	1.00	1.00
Yes	0.76 [0.56, 1.04]	0.77 [0.56, 1.07]
**Close friends**		
No	1.00	1.00
Yes	0.83 [0.58, 1.19]	0.91 [0.62, 1.32]
**Ghana**		
**Felt lonely**		
No	1.00	1.00
Yes	1.96*** [1.53, 2.50]	1.53** [1.19, 1.98]
**Peer support**		
No	1.00	1.00
Yes	0.78 [0.63, 0.98]	0.86 [0.68, 1.08]
**Close friends**		
No	1.00	1.00
Yes	0.84 [0.63, 1.12]	0.85 [0.63, 1.14]
**Liberia**		
**Felt lonely**		
No	1.00	1.00
Yes	1.30 [0.91, 1.87]	1.14 [0.78, 1.67]
**Peer support**		
No	1.00	1.00
Yes	0.88 [0.68, 1.14]	0.83 [0.64, 1.10]
**Close friends**		
No	1.00	1.00
Yes	0.77 [0.53, 1.12]	0.76 [0.52, 1.10]
**Mauritius**		
**Felt lonely**		
No	1.00	1.00
Yes	4.69*** [3.48, 6.31]	2.68*** [1.91, 3.75]
**Peer support**		
No	1.00	1.00
Yes	0.80 [0.62, 1.03]	0.94 [0.72, 1.24]
**Close friends**		
No	1.00	1.00
Yes	0.91 [0.61, 1.35]	0.82 [0.54, 1.25]
**Mozambique**		
**Felt lonely**		
No	1.00	1.00
Yes	1.45 [0.92, 2.28]	1.20 [0.74, 1.94]
**Peer support**		
No	1.00	1.00
Yes	0.73 [0.52, 1.01]	0.74 [0.52, 1.05]
**Close friends**		
No	1.00	1.00
Yes	0.48** [0.29, 0.81]	0.50* [0.29, 0.85]
**Namibia**		
**Felt lonely**		
No	1.00	1.00
Yes	1.93*** [1.56, 2.39]	1.57*** [1.25, 1.96]
**Peer support**		
No	1.00	1.00
Yes	1.06 [0.88, 1.28]	1.04 [0.86, 1.26]
**Close friends**		
No	1.00	1.00
Yes	0.97 [0.75, 1.26]	0.96 [0.74, 1.25]
**Seychelles**		
**Felt lonely**		
No	1.00	1.00
Yes	5.87*** [4.37, 7.88]	4.09*** [2.97, 5.64]
**Peer support**		
No	1.00	1.00
Yes	0.89 [0.67, 1.17]	0.87 [0.65, 1.17]
**Close friends**		
No	1.00	1.00
Yes	0.83 [0.54, 1.27]	0.83 [0.53, 1.29]
**Tanzania**		
**Felt lonely**		
No	1.00	1.00
Yes	2.36*** [1.65, 3.38]	1.94** [1.32, 2.85]
**Peer support**		
No	1.00	1.00
Yes	0.83 [0.65, 1.06]	0.91 [0.70, 1.17]
**Close friends**		
No	1.00	1.00
Yes	0.41*** [0.30, 0.56]	0.42*** [0.30, 0.58]

*Model I consisted of only the key explanatory variables

*Model II was adjusted for the covariates

## Discussion

Our study examined the associations between loneliness, social support and suicidal ideation among adolescents in SSA. We found that the prevalence of loneliness, peer support, one or more close friends, and suicidal ideation were 10%, 33.4%, 90.1%, and 14.5%, respectively. Namibia recorded the highest (14.9%) prevalence of loneliness while Tanzania had the least (6.4%). A possible reason for this finding of Namibian adolescents having the highest prevalence of loneliness in SSA could be as a result of the increased consumption of alcohol and other illicit drugs (e.g., tobacco) in the country [36]. It could also be that the substance use prevents formation of more meaningful social and peer relationships [36]. It has also been widely reported that negative peer influences can drive substance use and other mental health problems. While Liberia recorded the highest (20.7%) prevalence of suicidal ideation by the in-school adolescents, Tanzania recorded the least (12.0%). A possible explanation for this finding from Liberia could be the recent history of massive ethnic conflicts and exposure to traumatic violence in the country that have been widely discussed [37]. It has also been well documented that in conflict affected, humanitarian settings, intersectional violence especially sexual violence and displacement of families, communities can trigger suicidal ideation and both acute and long term psychological distress [38–40]. Evidence also suggests that mental health policies and programs are deficient in planning and poorly implemented in SSA countries. This is even more glaring in countries going through massive conflicts and humanitarian disasters [41, 42] and these could have accounted for the observed finding in our study.

The study found that in-school adolescents who felt lonely were more likely to experience suicidal ideation, a finding that was supported by other previous studies [15, 32, 43–45]. This finding could be attributed to the aggravation of the negative effects of psychological problems that are associated with having no one to share the problems one encounters [32]. This study provides additional empirical evidence to the second tenet of the IPTS which espoused that loneliness can trigger a reduction in belongingness which may result in some self-harming thoughts and behaviours [27]. This finding underscores the need to design and implement programmes that eliminate loneliness among in-school adolescents to help alleviate having thoughts of suicide. Essentially, making counselling services more accessible to all in-school adolescents without any form of restrictions would help in alleviating suicidal ideations.

However, in-school adolescents who had peer support had lower odds of having suicidal ideation. Some previous studies [15, 31, 32, 46] also reported similar findings. A possible reason for this observation could be a reflection of the indirect relationship between peer support and suicidal ideation [15, 31, 32]. It could also be that, since in-school adolescents have greater peer support or school or parental surveillance, they can share the problems they are facing with them for immediate solution, hence, reducing their likelihood of considering suicide [47]. This certainly provides credence for the second tenet of IPTS on which this study was premised [27]. This finding suggests the need for school authorities to create clubs or social groups in schools such as debate clubs, brigade and drama clubs, among others which will provide meaningful platforms for in-school adolescents to share and discuss issues of mental health to help alleviate having suicidal ideations whenever they encounter any problem.

In-school adolescents who had one or more close friends had lower odds of having suicidal ideation, suggesting the protective effect of having one or more close friends against suicidal ideation. Other previous studies in Mozambique[31], Nigeria [48] and Canada [49] have also confirmed this finding. It could be that those who had close friends are more likely to discuss their problems with their friends, reducing their likelihood of having suicidal ideation [50]. Our finding situates well with the IPTS framework that points to sense of belonging as a protective factor.

The study also found that in-school adolescents who felt lonely and lived in Benin, Ghana, Mauritius, Namibia, Seychelles and Tanzania were more likely to experience suicidal ideation. A possible reason could be that counselling services may be absent in many schools [51] in Benin, Ghana, Mauritius, Namibia, Seychelles and Tanzania, causing an increase in suicidal ideations among in-school adolescents. It could also be that mental health and counselling services in schools have been neglected or given less attention, increasing the risk of suicide behaviours as a result of bullying and other aggressive behaviours. This calls for urgent revamp in mental health and counselling services in schools in the identified countries in the SSA region.

### Strengths and limitations

The major strength of the study is the use of nationally representative survey data from the GSHS of eight countries in SSA. The questionnaires used in the GSHS helped to obtain quantitative data on all the variables of interest in this study. The large sample size obtained from systematic random procedure allows generalisability of findings to other homogenous populations. This study has some limitations that need to be acknowledged. First, the assessment of loneliness and suicidal ideation was based on self-reports, which are prone to recall and social desirability bias. Again, since the surveys employed cross-sectional study designs, it is difficult to attribute causation between loneliness and suicidal ideation. Finally, other socio-cultural and socio-economic factors that may contribute to suicidal ideation were not considered since they were not available in the GSHS datasets.

## Conclusion

The findings point to significant role of loneliness, and lack of social support, in understanding suicidal ideation. Countries in SSA need to improve child and adolescent mental health policies and programmes to respond to these risk factors and mental health challenges. It is also important to develop life-skills and psychosocial competencies in young people to promote social support, self-expression and support for common mental disorders so that suicidal ideation and attempts can be mitigated in time. It is important that countries in SSA advance child and adolescent mental health policies that address treatment, prevention and promotion in this population. Early screening and developing interventions for risk factors for suicidal ideation, including addressing experiences of loneliness and lack of social support needs prioritization to improve adolescent well-being in the region.

## Supporting information

S1 Table Study variables. (PDF)

## Abbreviations

cOR: Crude Adjusted Odds Ratio
aOR: Adjusted Odds Ratio
CDC: Centre for Disease Control and Prevention
CI: Confidence Interval
SSA: sub-Saharan Africa
VIF: Variance Inflation Factor
GSHS: Global School-based Health Survey
WHO: World Health Organization
