# Supplementary material for: In-school adolescents’ loneliness, social support, and suicidal ideation in sub-Saharan Africa: Leveraging Global School Health data to advance mental health focus in the region
Source: PLoS One. 2022 Nov 9;17(11):e0275660. doi: 10.1371/journal.pone.0275660 (PMC9645589; doi:10.1371/journal.pone.0275660)
Supplement: S1 Table — (PDF) [file pone.0275660.s001.pdf]

Table S1. Study variables

Variables	Question	Response options and recoding
**Outcome variable**		
Suicidal ideation	During the past 12 months, did you ever seriously consider attempting suicide?"	1 = yes, 2 = no<br>(Coded 2 = No; and 1 = Yes)
**Key explanatory variables**		
Loneliness	During the past 12 months, how often have you felt lonely?	1=never, 2=rarely, 3= sometimes, 4 = most of the time to 5 = always (coded as 1-3 = No; and 4- 5 = Yes)
Peer support	During the past 30 days, how often were most of the students in your school kind and helpful?	1=never, 2=Rarely, 3=sometimes, 4=most of the times, 5=always<br>(Coded as 1-3 = No; and 4–5 = Yes)
Close friends	How many close friends do you have?	1=0 to 4=3 or more<br>(Coded as 1=No; and 2-4=Yes)
**Covariates**		
Age	How old are you?	1=12, 2=13, 3=14, 4=15, 5=16, 6=17, 7=18 years (coded as 0=12-14, 15-19)
Sex	What is your sex?	1=male, 2=female<br>(Coded 2=Female, 1=male)
Hunger (proxy of socioeconomic status)	Went hungry past 30 days	1=never, 2=Rarely, 3=sometimes, 4=most of the times, 5=always<br>(Coded 1-3=No; and 4-5=Yes)
Tobacco use	During the past 30 days, on how many days did you use any other form of tobacco, such as chewing tobacco leaves?	1 = 0 days; to 7 = All 30 days<br>(Coded as 1 = No; and 2–7 = Yes)
Alcohol use	During the past 30 days, on how many days did you have at least one drink containing alcohol?	1 = 0 days; to 7 = All 30 days<br>(Coded as 1 = No; and 2–7 = Yes)
Cigarette smoking	During the past 30 days, how many days did you smoke cigarette?	1 = 0 days; to 7 = All 30 days<br>(Coded as 1 = No; and 2–7 = Yes)
Marijuana use	During the past 30 days, how many times have you used marijuana (country examples)	1=0 times; to 5=20 or more times (coded as 1=No; and 2-5=Yes)
Anxiety	During the past 12 months, how often have you been so worried about something that you could not sleep at night?	1 = never to 5 = always<br>(Coded 1 - 3 = No; and 4 – 5 = Yes)
Parents check homework (parental supervision)	During the past 30 days, how often did your parents or guardians check to see if your homework was done?	1=never, 2=Rarely, 3=sometimes, 4=most of the times, 5=always<br>(Coded as 1-3 = No; and 4–5 = Yes)
Understand problems (Parental	During the past 30 days, how often did your parents or guardians understand your problems and worries?	1=never, 2=Rarely, 3=sometimes, 4=most of the times, 5=always<br>(Coded as 1-3 = No; and 4–5 = Yes)

Connectedness		
Know what adolescent do free time (Parental or guardian Bonding)	During the past 30 days, how often did your parents or guardians really know what you were doing with your free time?	1=never, 2=Rarely, 3=sometimes, 4=most of the times, 5=always (Coded as 1-3= No; and 4–5 = Yes)
Parental or guardian respect for Privacy	During the past 30 days, how often did your parents or guardians go through your things without your approval?	1=never, 2=Rarely, 3=sometimes, 4=most of the times, 5=always (Coded as 1-3 = No; and 4–5 = Yes)
